# One-step metallization of weft-knitted fabrics for wearable biaxial strain sensors

**DOI:** 10.1038/s41598-022-24676-7

**Published:** 2022-11-21

**Authors:** Chao-Yi Tai, Chun-Yu Lin, Tang-Chun Liu, Lu-Chiang Jia, Thomas Jones, Amin Abdolvand

**Affiliations:** 1 Department of Optics and Photonics, National Central University, No. 300, Jhongda Rd., Jhongli Dist., Taoyuan, 320317 Taiwan; 2 Sousveillance Technology, Ltd., Taoyuan, 33858 Taiwan; 3 School of Science and Engineering, University of Dundee, Dundee, DD1 4HN UK

**Keywords:** Design, synthesis and processing, Sensors and biosensors, Laser material processing

## Abstract

One-step direct patterning of high definition conductive tracks in textiles is realized through laser direct writing in combination with a silver organometallic ink developed in-house. Photoreduction, nano-crystallization, and sintering are accomplished in one pass under the irradiation of a CW green laser light (λ = 532 nm) at moderate intensities (I ≥ 95 mW/mm^2^). By tailoring the surface tension and viscosity of the ink, high-definition conductive tracks are formed in weft-knitted polyester-Spandex composite fabrics, well-following the laser’s profile with negligible coffee stain effect. Length resistance as low as 4 Ω/cm is measured and anisotropy of the gauge factor as high as 25 is achieved. The metallized fabric exhibits reversible and hysteresis-free electromechanical responses subject to high strains. Durability assessment qualifies that the as-metallized strain sensors are able to sustain their performance for over 5000 stretch/release cycles, demonstrating its potential applications in biaxial strain sensing and interactive smart textiles.

## Introduction

Textiles integrated with electronics and photonics promise new avenues for realizing smart and interactive garments^1–3^. In parallel to the high demand of smart textiles, the substantial growth of internet of things (IoTs) market where everyday objects combine sensors, displays, computing, and communications, paves the way for enhancing functionalities on textiles^4,5^. The major industrial driving forces are the rapid development of advanced materials, nanotechnology, flexible electronics, and in particular, low-cost sensing technologies with cloud-accessibility. These new technological applications have enlarged the gamut of electronic device integration ranging from fashion and entertainment, sports and fitness, medical and healthcare, transportation, defense, and architectures^6^. To realize smart textiles, the cornerstone is to develop effective ways to pattern and turn the otherwise insulate fabrics into conductors.

Intrinsic conductive yarns (e.g., metallic wires) can be introduced into textiles by weaving^7^, knitting^8^, and embroidering^9,10^. In contrast, extrinsic means are capable of metallizing non-conductive fabrics directly via various deposition techniques^11–13^. The intrinsic method has advantages such as high conductivity, good ventilation, durability, and longer lifetimes. However, disadvantages of fewer material selectivity, poor flexibility, and relatively complex fabrication process, which all hinder electronic circuit patterning and scalable production as compared to extrinsic means. So far, the main applications for smart fabrics are limited to antimicrobial substances, anti-static and electromagnetic interference (EMI) reduction^14^.

With the rapid progress in nano-material synthesis, a large variety of electronic inks have been developed^15–18^ that promotes extrinsic means the mainstream methods for smart fabrics. The current trend is to use specific electronic inks in combination with chemical vapor deposition^19^, screen printing^20^, plating^21^, inkjet printing^22^, laser induced forward transferring (LIFT)^23^ and in particular, laser direct writing (LDW)^24^ to facilitate electronic circuitry patterning directly and masklessly on various substrates. For textiles, due to their porous nature, one-step direct patterning of high definition conductive path is still challenging. This is because of porous substrates such as fabrics made of fiber bundles are not flat and consist of many boundaries that makes the deposition of connective inks difficult. In addition, these boundaries scatter light and heat, which places further difficulty in reduction and sintering to make conductive pathways. Low temperature sintering methods which are applicable to a wide range of synthetic fabrics without damaging the consisting yarns are thereby urgently needed. This results in a persistent demand for developing low-temperature processible inks for rapid metallic ion reduction and post-deposition sintering^25^. Moreover, to broaden the applicability to textiles made of various materials and intercrossed structures, ink properties, in particular, the surface tension and viscosity should be tailorable for substrate wettability to infiltrate ink droplets into yarns and between their junctions. It is also important to balance the surface tension and evaporation rate of the ink to mitigate coffee ring effect which is a signpost of uniformity. These requirements rule out particle-based inks and render particle-free reactive organometallic inks the best candidate to achieve high-definition conductive patterns on textiles with desirable stretching capabilities. Besides, to make strain sensors based on metallized fabrics, enhanced adhesion that is immune to large stretching/releasing cycles is also important.

In this paper, we describe the fabrication of high definition conductive tracks on weft-knitted polyester-Spandex blended textiles based on a homemade reactive organometallic silver complex^26^. A modified Tollen’s process is adopted which is designed for one-step laser direct metallization. Essentially, the formulation is composed of three parts: the silver precursor, solvents, and additives. Combining established but yet to be integrated knowledge, the rationale of present study is described as follows. Silver oxide (Ag_2_O) and ammonium carbonate (NH_4_)_2_CO_3_ are chosen as the precursors because of their high stability and capability of producing the minimal organic residuals and impurities^27–31^. Dibutylamine and ethylene glycol are used as solvents that not only facilitate solubility to promote the formation of [Ag(NH_3_)_2_]^+^ cations, but behave as moderate reductants to improve film connectivity. To lower the processing temperature and balance Marangoni and convective flows^32^, isopropyl alcohol and glycerol are used as additives. In addition, polyethylene terephthalate (PET) fabrics are pretreated with sodium hydroxide that introduces and activates the so called alkaline hydrolysis process. This not only generates ethylene that acts as an initiator to reduce [Ag(NH_3_)_2_]^+^cations^33^, but creates nano-pits on the surface of the PET fabrics to enhance Ag adhesion. By adjusting the laser parameters with surface tension and viscosity of the ink tailored for PET fabrics, excellent infiltration and high definition conductive tracks are successfully made in one single step at low temperatures. The preparation of the reactive organometallic silver complex is presented in the Methods.

## Results and discussion

**Figure 1:**
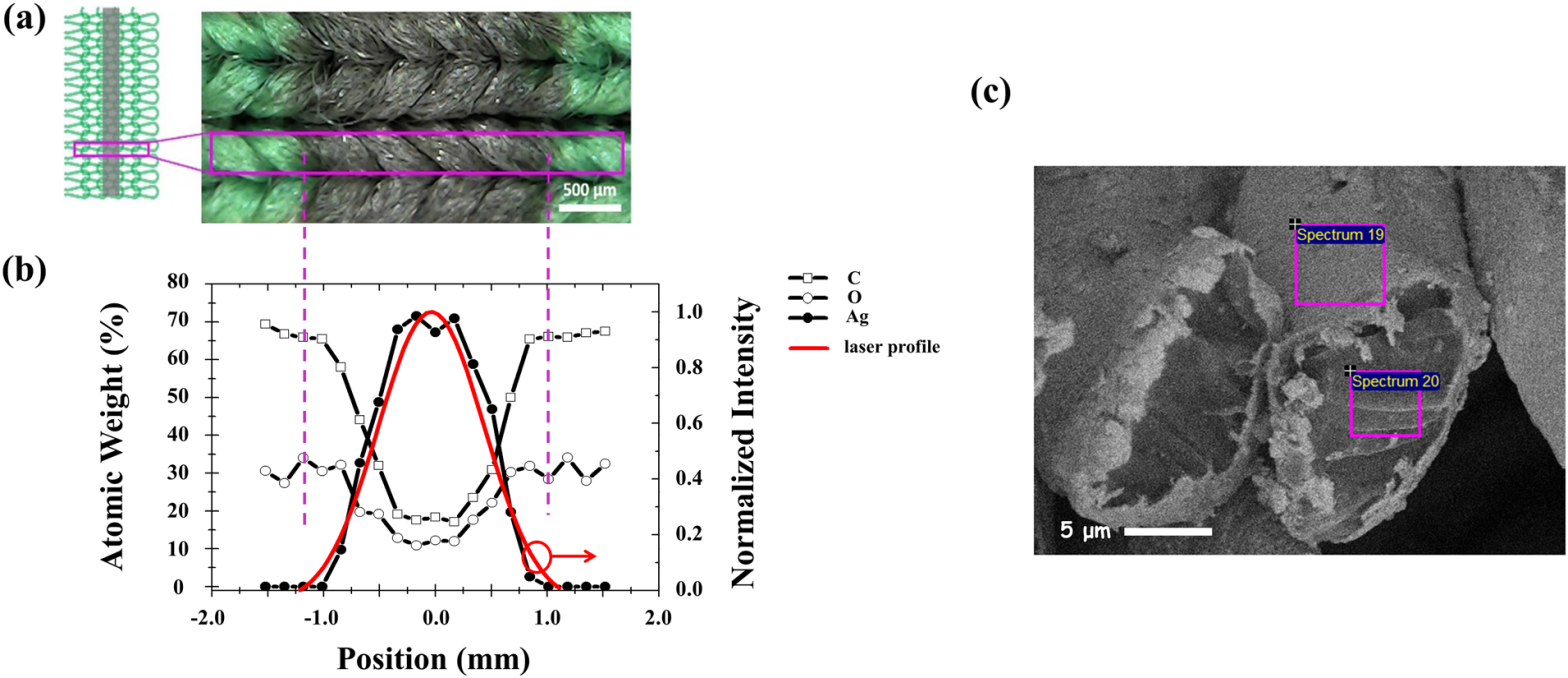
(**a**) Optical image of a typically metallized weft-knitted fabric where the dark region is Ag rich with high definition edges. (**b**) Relative atomic weight of elements C, O, and Ag measured by EDS. Also shown is the normalized laser intensity which correlates closely with the distribution of Ag element. (**c**) SEM image of the cross-section of the as-metallized fabric.

Prior to deposition, opto-thermal properties of the synthesized ink are studied. A droplet of the ink, hung at the tip of a needle, is irradiated by the laser. Its image and temperature are monitored by an infrared (IR) camera (Fig. S1, Supplementary Information). It is found that the initially transparent droplet turns darker gradually, and eventually becomes opaque with the increasing time of irradiation. In parallel to this, the measured temperature exhibits 2 plateaus corresponding to the onset of silver nanoparticle formation, and the agglomeration into bigger flakes, respectively. With the growing of bigger metallic particles, scattering and reflection gradually dominate over absorption that prohibits further increase of temperature. Until stable, the highest temperature measured is no higher than 60 °C, demonstrating the capability for low temperature processing on PET fabrics. Figure 1a shows the optical image (OM) of a typical conductive path fabricated on weft-knitted PET textiles. High definition Ag coated trace (in black color) is clearly in evidence. Having this fabrication capability on textiles is of substantial importance, in particular, for inductors and antenna patterns where the impedance and operational frequency depend sensitively on printed geometrical shapes. The spatial distributions of three major elements C, O, and Ag measured by energy dispersive X-ray spectroscopy (EDS) are depicted in Fig. 1b. It is found that the relative atomic weight of Ag follows closely with the energy profile of the laser light. Their overlapping is estimated to be better than 95%, indicating an excellent combination of ink formulation and LDW parameters. This result renders LDW a powerful tool that makes the laser itself behaves as a ‘pen’ to pattern fabrics with high fidelity. Figure 1c shows the SEM image of the cross-section of the as-metallized fabric. It is clear in evidence that metallization occurred effectively not only on the surface but between fibers.

**Figure 2:**
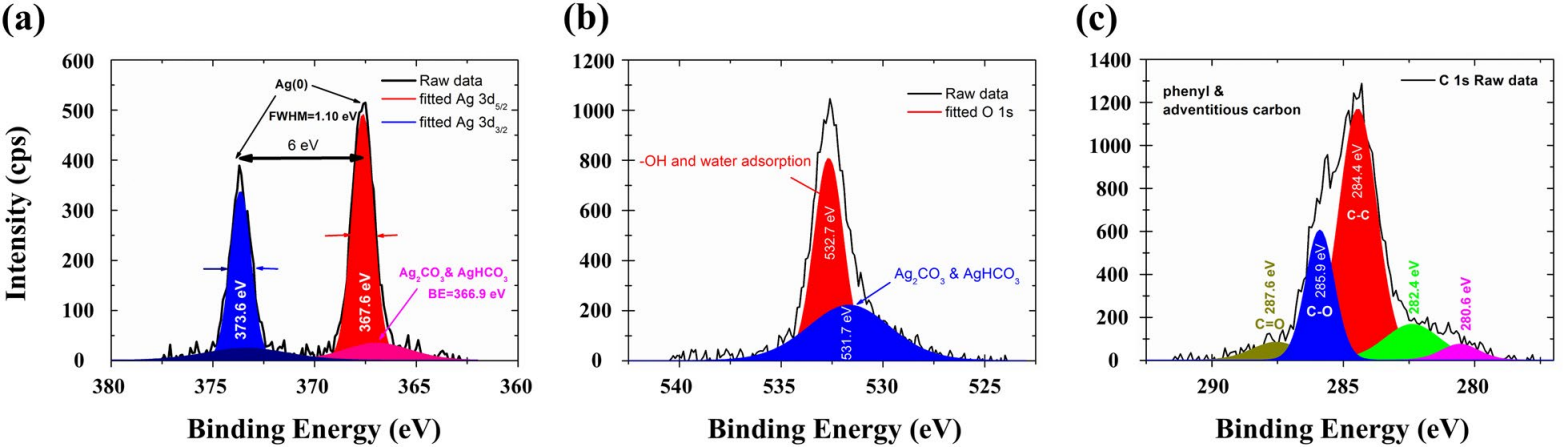
X-ray photoelectron spectroscopy of (**a**) Ag 3d, (**b**) O1*s*, and (**c**) C1*s* states on the surface of the as-metallized PET fabric. Fitting is carried out using software CasaXPS which is detailed in Methods.

To analyze the chemical states of the surface finishes, X-ray photoelectron spectroscopy (XPS) is used to characterize the relative ratio of deposited species. As shown in Fig. 2a, the Ag 3d spin–orbit split peaks at binding energies (BEs) of 367.6 eV (Ag 3d_5/2_) and 373.6 eV (Ag 3d_3/2_) are identified. The evidence that the separation between the two peaks (6 eV) and the full-width half-maximum (fwhm) bandwidth (1.10 eV) strongly suggest the measured BEs originate from metallic state of Ag^34^. CasaXPS fitted result also shows that both peaks can be fitted predominantly by a single Gaussian–Lorentzian peak along with a broad and much weaker peak, assigning to silver carbonate and silver hydrocarbonate^35^.

Consistent results can be found in O1*s* and C1*s* XPS spectra where BEs peaked at 531.7 eV and 287.6 eV correspond to oxygen containing carbon species, as shown in Fig. 2b,c. This result is in close agreement with those published in literatures^34,35^. Apart from this, the position dependent spectra (Fig. S2, Supplementary information) indicate that the metallization process is sensitive to the processing temperature as well. Away from the center of the conductive path, signals from impurities such as residues of incomplete reactions, adsorbed water, carbonate, hydrocarbonate, and adventitious carbon are found. This is evidenced by the decreased intensity of the Ag 3d_3/2_ and Ag 3d_5/2_ peaks and the presence of the peaks located at lower BE side of Ag 3d and O1*s* peaks, respectively. The relative weight ratio between Ag and impurities across the conductive track reveals the level of reduction that can be viewed as an effective indicator for the resulting conductivity. Taking the intensity ratio between the Ag 3d_5/2_ and Ag_2_CO_3_ and AgHCO_3_ peaks in XPS spectrum (Fig. 2a), the level of reduction is estimated to be more than 95%., indicating the reduction is nearly complete and the production of residues from the precursor is less than 5%. The slight shift of the Ag 3d_5/2_ peak towards lower BEs implies that smaller Ag clusters are primarily formed in the middle of the track and bigger clusters are mostly formed at the edge^36,37^. The abovementioned phenomena can attribute to the temperature gradient across the laser spot as well as the “self-sorting” effect due to the plasmonic mediated trapping force^38^, as described below.

**Figure 3:**
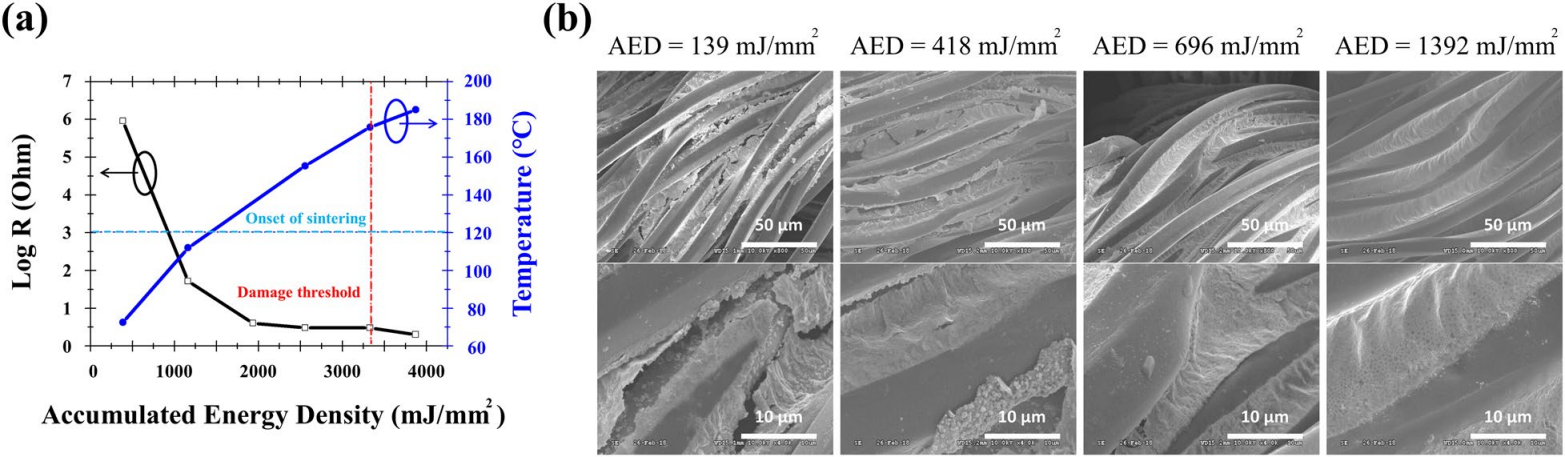
(**a**) The length resistance and real-time temperature as a function of the accumulated energy density (AED). (**b**) SEM images for fabrics treated with various AED. The fabric surface finish turns smoother and void-free when the AED is increased beyond 1392 mJ/mm^2^.

To optimize the conductivity and to avoid damage to the fabrics, combinations of fabrication parameters were investigated including powers of the laser *P*, spot size *d*, translational speed *v*, and scan times *N*. These variables are then converted into the so-called “dose” or the accumulated energy density (AED) used here via Eq. (1)^39^which acts as a single parameter to characterize the fabrication conditions. It should be noted that the coefficient 0.76 here considers the realistic energy distribution as a Gaussian profile. Figure 3a shows the length resistance (resistance per centimeter) and the instantaneous temperature upon processing as a function of the AED. Essentially, the log-scaled resistance decreases with the increase of the AED. As soon as the AED reaches 2000 mJ/mm^2^, the length resistance is reduced significantly from several MΩ/cm to R = 4 Ω/cm. This is a typical behavior of reaching percolation threshold when the accumulated thermal energy causes effective sintering. The corresponding temperature T = 120 °C at AED = 1392 mJ/mm^2^ is considered as the onset of sintering that is evidenced by the SEM images described in Fig. 3b. Further increases the AED beyond 2000 mJ/mm^2^ results in almost no change of the electrical resistance, and the lowest value found is R = 4 Ω/cm. When the AED is increased above 4000 mJ/mm^2^, the fabric is damaged. The IR camera measured temperature (T > 180 °C) is reasonably in close agreement with the melting point of synthetic polyesters.

1 $$AED = 0.76 \cdot \frac{4P}{{\pi \cdot d \cdot v}} \cdot N$$

The morphological evolution is characterized by the SEM images taken for fabrics subject to successive AEDs. As shown in Fig. 3b (upper row), with the increase of AEDs, the filling fractions of Ag nanoparticles (NPs) increase, and the surface of PET fabrics turns smoother. This is attributed to the plasmon assisted sintering effect. Namely, reduction and nucleation are firstly triggered by laser irradiation. With the growing of the size of NPs, they can no longer be trapped by the gradient force of the laser beam. As a result, bigger NPs are propelled alongside the fibers due to convection^40^. This “self-sorting” mechanism is originated from size dependent plasmon resonance^41^ and is clearly in evidence from the SEM images, wherein, bigger NPs are accumulated aside. The magnified SEM images (Fig. 3b, lower row) show the degree of sintering in detail. When AED = 418 mJ/mm^2^, no sintering is observed. It is not until AED = 1392 mJ/mm^2^ that the sintering is complete. Under this circumstance, a film-like conductive layer forms. Further increase the AED results in pinholes or voids which are likely due to the evaporation of solvents, as a result of rapid temperature rise from plasmonic hot spots.

The strain sensing characteristic is evaluated by analyzing the electro-mechanical responses in both the course and wale directions. The as-metallized fabrics are tested under strains range from ε = 0% to 100%. It is found that after stretching/releasing the as-metallized textiles for over 50 cycles at a maximum strain of ε = 100%, the resistance shows negligible hysteresis, implying the electro-mechanical response is no longer dependent on fabric’s stretch/release histories. For the sake of consistence and repeatability, experiments are then conducted with fabrics pre-trained subject to abovementioned process.

The change of electrical resistance as a function of the applied strains exhibits a bell-shaped composite-like characteristic^42,43^, i.e., the resistance rises with the increase of the strain followed by a subsequent decrease of resistance. This behavior indicates that the intrinsic mechanical property of weft knitted fabrics is preserved. It should also be noted that a strain range of 40% is sufficient to cover most of the human motions. The bell-shaped electromechanical response presents an additional advantage to distinguish low/high strain loads basing on such bistable characteristic. By contrast, most textile-based strain sensors to date exhibit only monotonically decreased or increased resistance over very limited working strain range. Without comparing the properties over the entire strain range, one cannot judge whether the conductive coating affects the intrinsic property (e.g. air permeability and elasticity) of the undoped materials.

**Figure 4:**
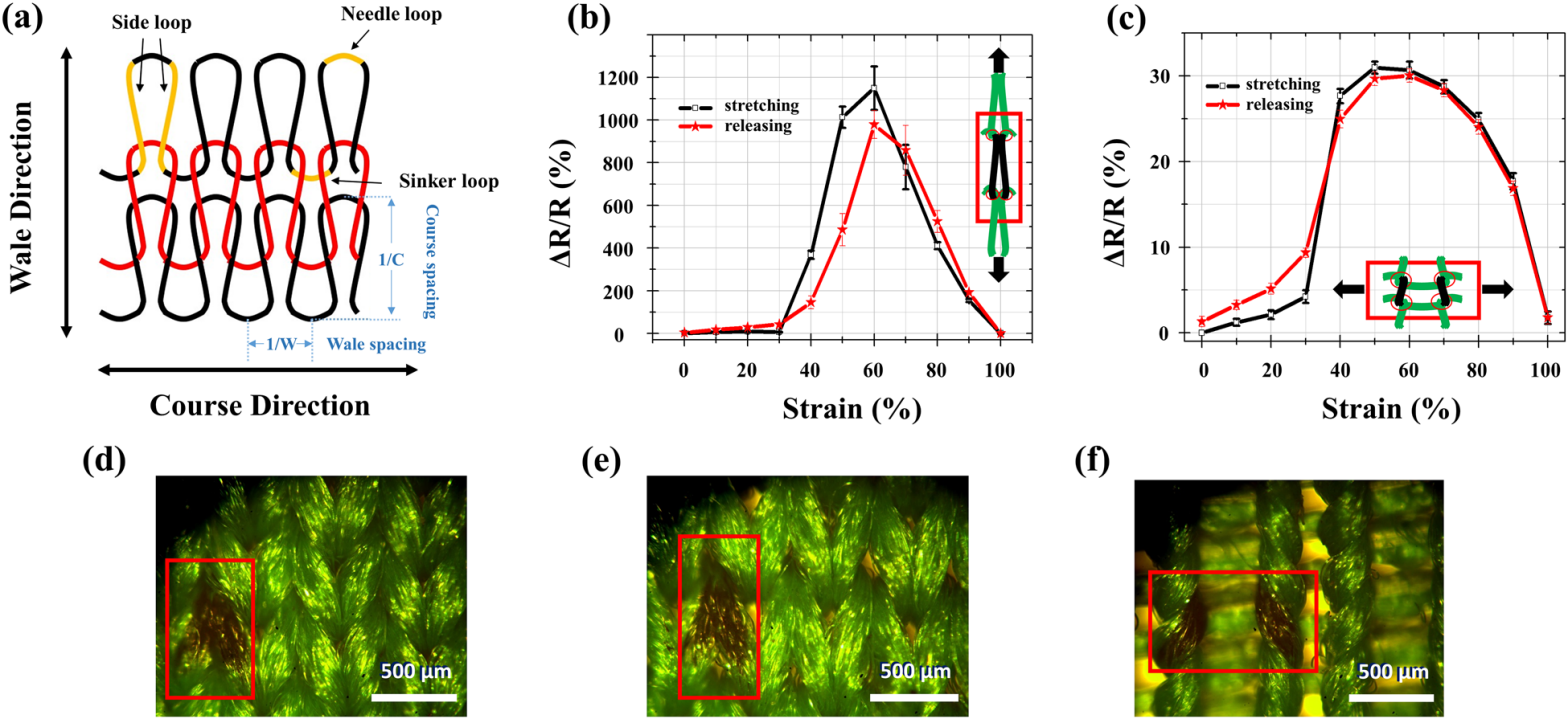
(**a**) Schematic view of a typical weft-knitted textile where the repeated unit cell (RUC) is composed of two side loops, a needle loop, and a sinker loop. (**b**) Relative variation of resistance as a function of the applied strain in the wale direction and in the course direction (**c**). (**d**–**f**) Optical image of the un-stretched, wale-direction stretched, and course-direction stretched fabrics, respectively. The anisotropic deformation of the RUC is clearly in evidence as depicted in the insets of (**b**) and (**c**).

Figure 4a shows schematically the repeated unit cells (RUCs) of the weft-knitted fabrics. The electromechanical responses in the wale and course directions are shown in Fig. 4b,c, respectively. The initial increase of resistance is due to the breakdown (elongation and slippage between yarns) of the otherwise connected silver clusters, which results in the reduction of conductive pathways. Subsequent decrease of resistance at higher strains is attributed to the orientation effect^42^ along with the structural deformation which will be explained in due course. The orientation effect is caused by re-orientation of the deposited clusters which tend to align in the direction of the strain applied. Like most elastomeric yarns which inherently differ from rigid fibers, the effective distance between isolated clusters decreases upon elongation. Until percolation, the resistance reaches the minimum^44^. Apart from the orientation effect, the structure induced anisotropic electro-mechanical responses between the course and wale directions are also noteworthy. The gauge factor (GF), defining by the ratio of relative resistance variation ∆R/R to the mechanical strain ε (Eq. 2), is 25 times larger in the wale direction (GF = 19.15) against that in the course direction (GF = 0.77). Such a large anisotropy promises substantial applications in monitoring motions of twist or torsions.

2 $$GF = \frac{\Delta R}{{\varepsilon R}}$$

Compare to silicon and single material (non-composite) based strain sensors^45^, the present study shows superior result. A comprehensive review of the reported GFs can be found in the reference^46^. Such anisotropy was also observed in published result^47^ however it was only tested under very low strains (ε < 10%) and the physical mechanism behind has yet to be addressed. It is justified here by considering the geometrical structures of weft-knitted fabrics. Figure 4d shows the optical microscope (OM) image of un-stretched fabrics. Upon stretching along the wale direction as schematically shown in Fig. 4b (inset) and in Fig. 4e for the corresponding OM image, the effective lengths of both the needle loop and the sinker loop decrease while that of the side loop increases. For the cases where fabrics are subjected to high strains (ε > 50%), the adjacent RUCs deform into highly elliptical shapes and the side, head, and sinker loops contact successively. Eventually, the RUCs are no longer consisting of interpenetrated circular loops but segments of straight lines (or bundles). According to Holm’s theory^48^, the contact resistance *R*_*c*_, playing a dominant role to dictate the overall resistance of the equivalent circuit of weft-knitted RUCs, is determined by Eq. (3):

3 $$R_{c} = \frac{\rho }{2}\sqrt {\frac{\pi H}{{nP}}}$$

where *ρ* is the resistivity of the metalized polyester yarn, *H* represents material hardness, *P* is the contact pressure, and *n* stands for the total number of contact points. The significant increase of contact points and pressure under high strains account for the reduction of overall resistance, leading to negative GF variations. By contrary, when stretching along the course direction as schematically shown in Fig. 4c (inset) and in Fig. 4f for the corresponding OM image, the number of contact points remains and the consisting loops, i.e. the needle, sinker, and side loops between adjacent RUCs are all well separated. Under the same circumstance, it is merely the increased contact pressure *P* which causes the moderate decrease of the resistance. It is not until the needle and sinker loops contact at high strains that the overall resistance decreased. It should be noted that the applied strain here is characterized by the percentage elongation of the fabric length. According to reference^49^, widthwise stuck sooner for weft-knitted textiles. Since the wale density (i.e., inverse of the wale spacing, also known as the wale number.) in the present case is W = 17.54 cm^−1^ which is larger than the course density C = 32.26 cm^−1^. This structural asymmetry results in higher contact pressure^50^ (or reaction force) and therefore larger change of resistance in the wale direction at the same elongations. It is worth commented here that the bell-shaped electromechanical responses (Fig. 4b,c) agreed qualitatively with the stress–strain curve of the uncoated PET yarns^51^. The anisotropy of the gauge factors between wale (GF = 19.15) and course (GF = 0.77) directions is also agreed with the simulated reaction force versus strain curve^50^ of like materials. These evidences indicate that the metallized fabric still keeps the intrinsic mechanical properties of the uncoated fabric, which is a property seldom addressed by most of the research to date.

**Figure 5:**
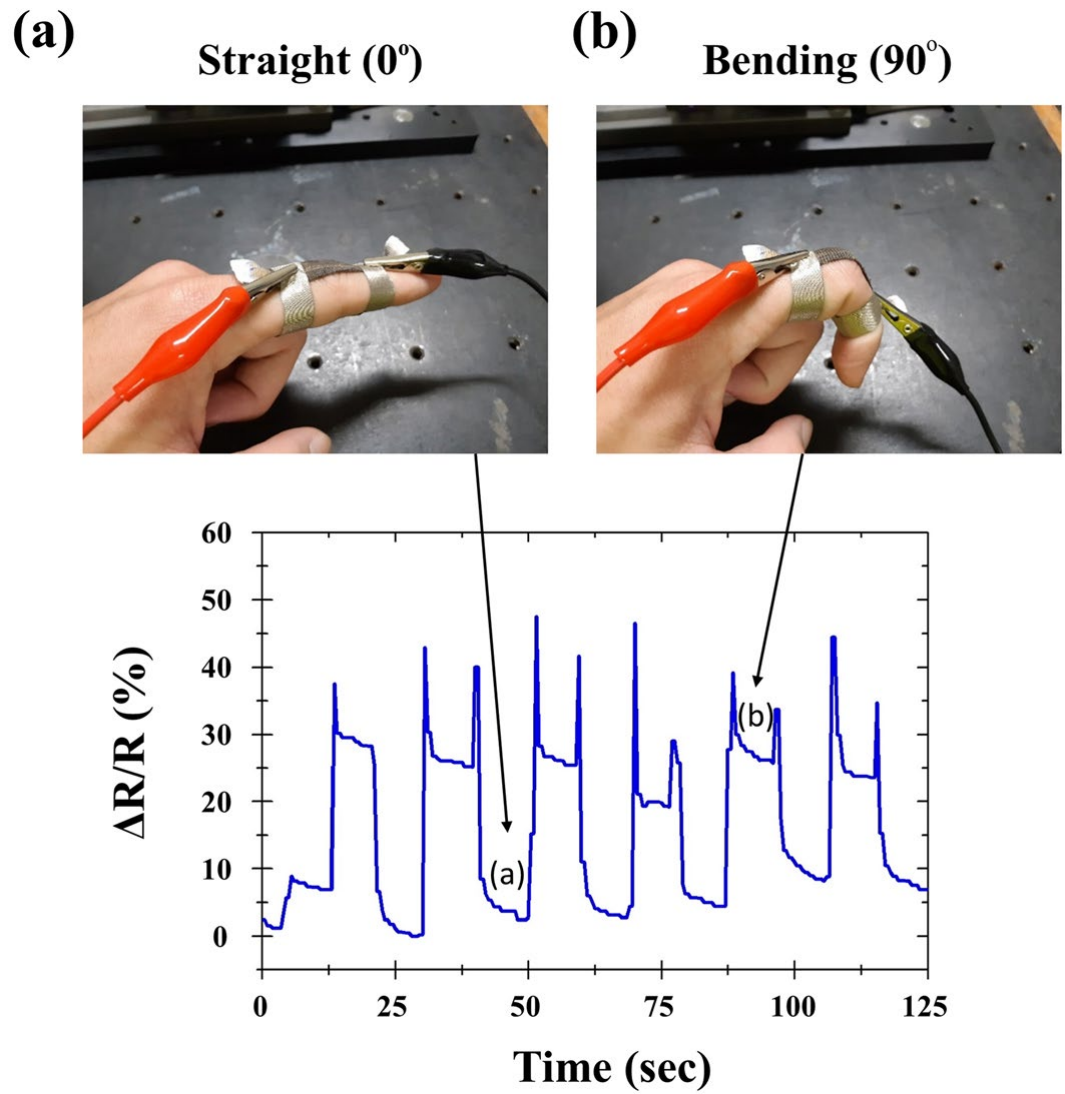
Illustration of a textile based strain sensor for human motion sensing. The contrast of relative resistance variation between finger bending at 0*°* (**a**) and 90*°* (**b**) is over 25. The spikes are due to instabilities of human factors and the nonlinear response upon sudden motion changing. Similar observations were also made in^52–54^.

**Figure 6:**
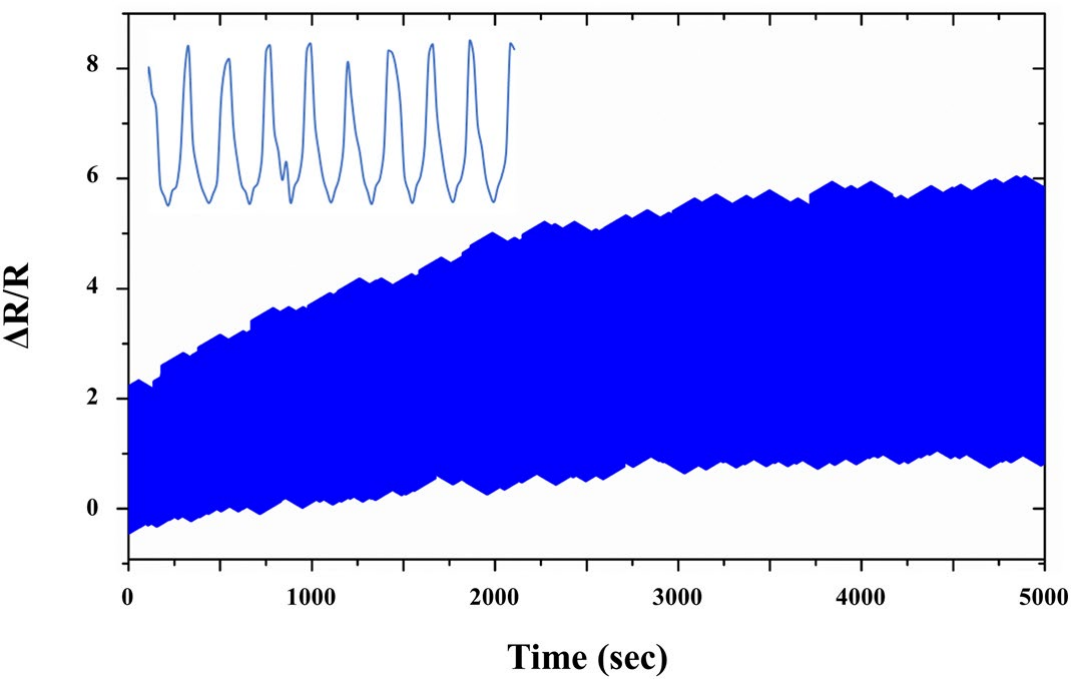
Relative resistance change under repeated strains between *ε* = 0–10% at 1 Hz.

Finally, to demonstrate the application for human motion sensing, the detection of finger bending is illustrated. As shown in Fig. 5, the as-metallized fabric is attached along the index finger and over 25% resistance variation is detected between the straight state (finger bending at 0°) and the bending state (90°). This performance is comparable to current state of the art^43^ while the fabrication is much simplified. The spikes are attributed to human factors as well as the nonlinear response produced each time the sensor was subjected to sudden changes. Similar behavior was also observed and explained^52–54^. The durability of the as-metalized textiles is also assessed, as shown in Fig. 6. The experimental result reveals that under a dynamic scan of strains between ε = 0–10% at 1 Hz, the relative resistance change gradually reached a stabilized response after 5000 cycles. This result shows that the as-metallized yarns can be further optimized by protective coatings, as in many other studies. Nevertheless, the present result already provides a ready scaffold for wearable sensors and intelligent textiles.

## Conclusion

We obtain a wearable strain sensor based on weft-knitted polyester-Spandex blended fabrics via laser direct metallization of silver-based organometallic reactive ink. The electromechanical response to strains applied in the wale direction is 25 times larger than that in the course direction, rendering its potential application for biaxial sensing. The success of producing high-definition conductive path with a high degree of overlapping between Ag distributions and the laser energy profile signifies the excellent combination between the formulated electronic ink and LDW technique. This study provides a shortcut solution for circuitry-on-textiles whenever there is a need for rapid prototyping of complex patterns with high precisions. Besides, the strain sensor presents negligible hysteresis, high gauge factors, broad strain sensing range, good repeatability, and excellent durability against large stretching/releasing cycles, which altogether sheds light on interactive and smart textiles.

## Methods

### Formulation of organometallic complex

Chemicals of analytical grade are obtained from Sigma-Aldrich without further purification. The precursor is prepared by dissolving silver oxide and ammonium carbonate with molar ratio 1:2 in N-butylbutan-1-amine which is pre-mixed with ethane-1,2-diol with molar ratio 1:3 at 25 °C. After sonication for 10 min followed by centrifuging at 500 rpm for 1 h, the precursor is allowed to pass a syringe filter with pore size 0.2 µm (Pall Life Science). Finally, small amount of propane-1,2,3-triol is added, adjusting the surface tension to a value of σ = 26.7 ± 0.3 mN/m which is smaller than the surface free energy of PET (σ = 44 mN/m)^55^. This choice enhances ink spreading over the yarns that yields the lowest length resistance combing with the optimized laser parameters. The flowchart of developing the organometallic ink is schematically shown in Fig. S3.

### Surface tension measurement

The surface tension of the synthesized organometallic complex is measured by the optical pendant drop method where a drop of ink with volume V = 3.4 µL is hanging from a syringe needle. By analyzing the optically magnified droplet profile (KRÜSS DSA 100), the principle radii of a curved section (Area = 10 mm^2^) of the droplet are determined. These parameters are in turn used to determine the value of surface tension with the so-called fundamental equation for a pendant drop in hydromechanical equilibrium^56^.

### Metallization of fabrics by LDW

Synthetic textiles consisting of 93 wt% polyethylene terephthalate blended with 7 wt% Spandex are obtained from Sousveillance Ltd. The composition is the same as typically off-the-shelf garments. Before metallization, the fabrics are washed sequentially in deionized (DI) water, ethanol, and DI water again. After drying in an oven, the fabrics are immersed in 5 M NaOH at 60 °C to create nanopits which are used to enhance the adhesion of Ag clusters to be reduced afterward. Due to alkaline hydrolysis, ethylene glycol (EG) is generated that plays crucial roles as an initiator, and also known as a moderate reducing agent^57^ providing better control of the size of NPs generated. Subsequently, the formulated ink is drop coated onto the pre-treated fabric, where the drop volume V = 10 µl and droplet spacing D = 5 mm are controlled by a syringe in combination with a translational stage. Immediately after drop coating, the continuous wave green laser (Millennia, Spectra-Physics, CW, λ = 532 nm) is directed to the region where ink droplets applied onto. By translating the laser spot (2.54 mm $\times$ 1 mm, FWHM size) along the track of ink on the fabric at a speed of v = 0.125 mm/s, conductive paths can be obtained in a single scan with an accumulated energy density (AED) over 2000 mJ/mm^2^ that yields a length resistance as low as 4 Ω/cm. It should be noted that during the scanning of the laser light, an infrared (IR) camera (FLIR One Pro) is used to monitor the temperature of the fabric in real-time. The highest temperature recorded is 150 °C which is well below the damage threshold of PET fabrics.

### EDS and XPS characterization

EDS measurement is carried out using a field emission scanning electron microscope equipping with an X-ray energy dispersed analyzer (Hitachi SU-8010). Setting the acceleration voltage to 15 kV, the achievable spatial resolution is 1 nm with a penetration depth of 1 µm. The atomic weight ratio between C, O, and Ag elements are measured for the front, back and cross-sectional surfaces. For the XPS measurement, monochromatic X-ray generated from Al Kα line with an energy of 1486.6 eV and a spot size range from 15 to 400 µm are used as the excitation. The ejected electrons from surfaces of the fabrics are dispersed by a hemispherical sector analyzer (Thermo VG Scientific) in constant analyzer energy (CAE) mode, which gives a theoretical energy resolution of 20 meV. The obtained data are analyzed with processing software CasaXPS^58^. In brief, a Shirley background and line shape composed by Gaussian/Lorentzian with relative fraction of 30/70 are used for deconvoluting and fitting the measured photoemission peaks. Database from NIST X-ray photoelectron spectroscopy of Ag and silver oxides is used^59^ and the residual standard deviation is minimized to obtain the best fitting of experimental data.

### Resistance measurement

According to the standard ASTM D257-07^60,61^, the length resistance is measured by four-point-probe method where a current source and voltmeter are used to derive the resistance. Electrodes made of Cu conductive tapes are stuck on the surface of the fabrics with a spacing of 1 cm. The fabrics under study are fixed on a computer controlled translation stage, enabling real-time resistance measurement upon stretching/releasing cycles.

## Supplementary Information

Supplementary Information.

## Data Availability

The datasets used and analyzed during the current study available from the corresponding author on reasonable request.
