# Supplementary material for: One-step metallization of weft-knitted fabrics for wearable biaxial strain sensors
Source: Sci Rep. 2022 Nov 21;12:20029. doi: 10.1038/s41598-022-24676-7 (PMC9681773; doi:10.1038/s41598-022-24676-7)
Supplement: Supplementary file 1 — Supplementary Information. [file 41598_2022_24676_MOESM1_ESM.pdf]

Supplementary information

One-step Metallization of Weft-knitted Fabrics for Wearable Biaxial Strain Sensors

Chao-Yi Tai[1,*], Chun-Yu Lin[1], Tang-Chun Liu[1], Lu-Chiang Jia[2], Thomas Jones[3], and Amin Abdolvand[3]

[1]Department of Optics and Photonics, National Central University, Taoyuan, 320317, Taiwan
[2]Sousveillance Technology, Ltd., Taoyuan, 33858, Taiwan
[3]School of Science and Engineering, University of Dundee, Dundee, DD1 4HN, UK
[*]cytai@dop.ncu.edu.tw

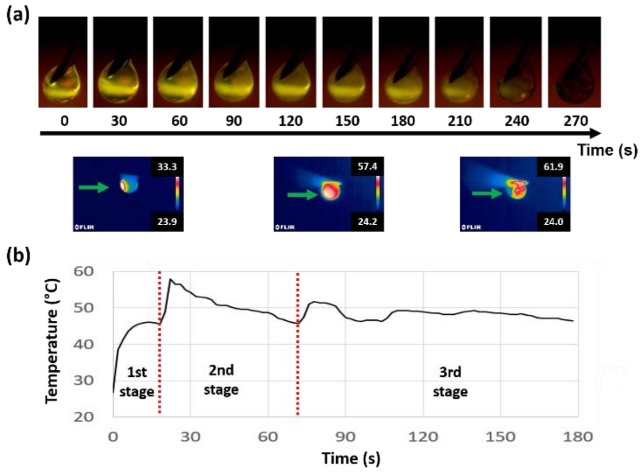

Figure. S1. (a) Optical images of ink droplets hung on the tip of a needle upon laser irradiation. Light streak is clearly seen and the originally transparent droplet turns opaque gradually. The images of the IR camera show that initially the hot spot is localized on a small portion of the droplet surface. With the increase of irradiation time, the hot spot is enlarged andeventually its shape no longer follows that of the droplet. The presence of irregular shaped hot spot is a signature of forming larger clusters or flakes of silver. (b) The instantaneous temperature measured as a function of irradiation time. The plateaus during the 1*st* and 2*nd* heating stages are attributed to nucleation and agglomeration of silver nanoparticles, respectively. The 3*rd* stage presents clusters and flakes formation that causes larger agitation upon thermalization with environment. Note that the recorded temperature here is no higher than 62 °C implying the low temperature processability.

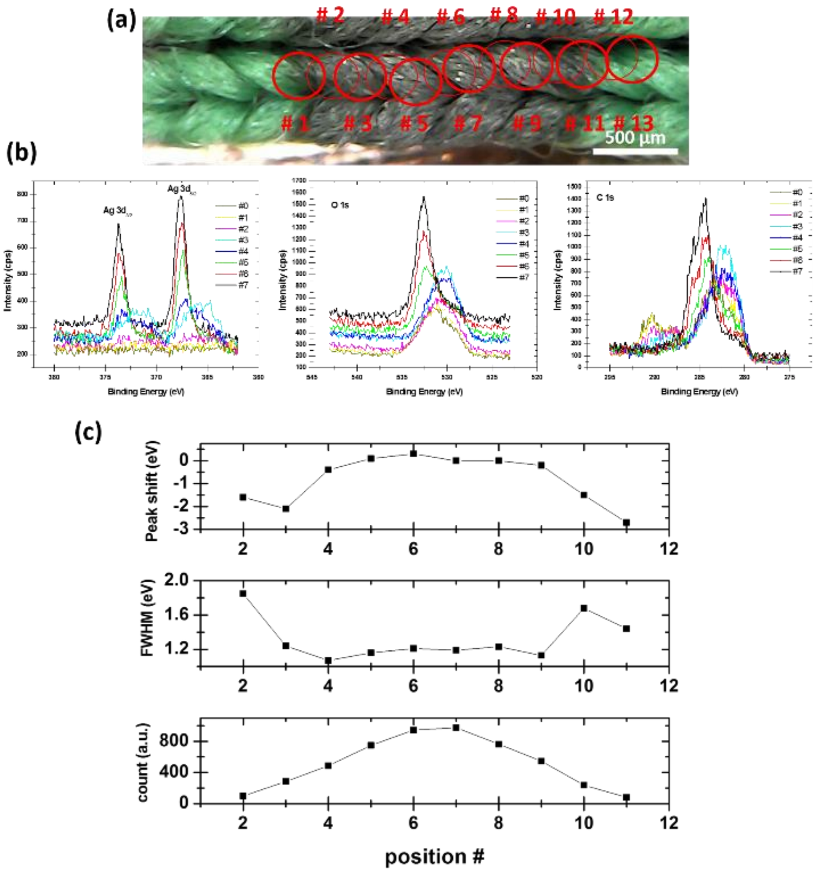

Figure. S2. (a) A schematic view of regions where XPS are measured. The red circles depict the positions of the X-ray spots which are 200 μm in diameter. (b) Raw data of the position dependent XPS results for Ag 3d, O 1s, and C 1s states. Note that position #7 located in the middle of the laser spot and position #0 located just outside of the laser spot. (c) Away from position #7, not only the peak intensity of Ag 3d drops but the position down-shifts to lower BEs. Besides, the fwhm of the measured peaks increases with the distance away from the middle, indicating the presence of impurities. Similar results are obtained in O 1s and C 1s states. This result is obtained with an X-ray spot size of 300 μm.

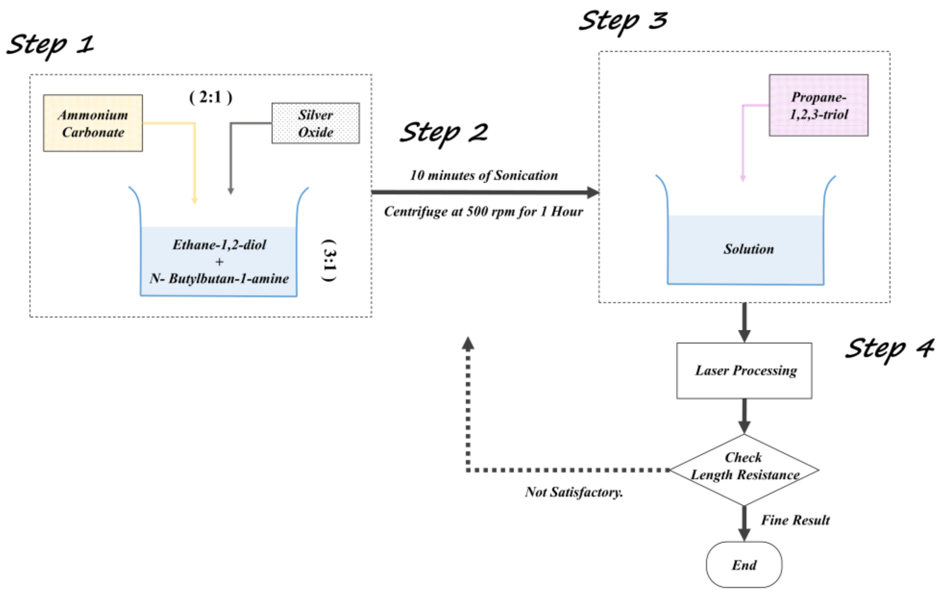

Figure S3. Flowchart of the ink synthesis process.
